# Topical Promethazine Side Effects: Our Experience and Review of the Literature

**DOI:** 10.1155/2013/151509

**Published:** 2013-11-19

**Authors:** C. Cantisani, S. Ricci, T. Grieco, G. Paolino, V. Faina, E. Silvestri, S. Calvieri

**Affiliations:** ^1^Department of Dermatology and Plastic Surgery, University “Sapienza” of Rome, Viale del Policlinico 155, 00161 Rome, Italy; ^2^Section of Legal Medicine, Department of Anatomical, Histological, Medical Legal and Locomotive System Sciences, University of Rome “Sapienza”, Viale Regina Elena 336, 00161 Rome, Italy

## Abstract

Promethazine hydrochloride is a first-generation H1 receptor antagonist, antihistamine, and antiemetic medication that can also have strong sedative effects. The apparent ability of topical H1r/2r antagonists to target epidermal H1/2r was translated into increased efficacy in the treatment of inflammatory dermatoses, likely due to decreased inflammation and enhanced barrier function.

## 1. Introduction

The elderly represent the fastest growing segment of the population all over the world, and the most common skin complaint in this age group is pruritus, due to a multitude of variables (physical and cognitive limitations, multiple comorbid conditions, and polypharmacy) [1]. To date, there is no universally accepted therapy. Currently, management of pruritus in the elderly must take an individualistically tailored approach with consideration of the patient's general health, the severity of symptoms, and the adverse effects of treatment. Most patients cannot deal with their pruritus without taking antihistamines (AHs) daily [2]. In fact, the use of topical AH has been reported to have an immediate effect; it reduces pruritus significantly in patients with eczematous dermatitis, xerotic skin, or insect bites. It was shown that single application of topical AH suppressed histamine for at least 2 h [3]. Widespread use of self-medication, low-cost, and nonprescribing approach leads to a diffusion of these topical agents, especially in Italy, in patients with insect stings, pruritus, or solar burns. Topical AH has been reported to have a quick onset of action in relieving eczema-associated pruritus, but it has a short duration of action inducing patients to use it several times per day. Similar to histamine, AHs have organ-specific efficacy and adverse effects [4].

## 2. Promethazine

**Figure 1:**
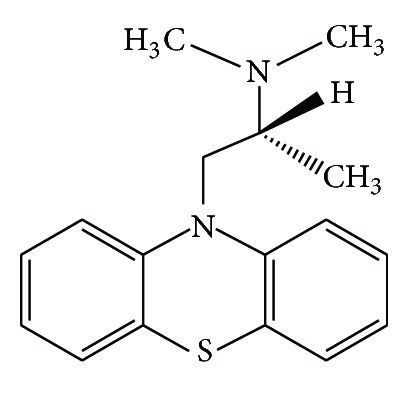
Promethazine structure.

An antihistamine should ideally possess high selectivity for the H(1)-receptor, high PrH1R occupancy, and low to no BrH1R occupancy. Promethazine (PM) ((*RS*)-*N*,*N*-dimethyl-1-(10*H*-phenothiazine-10-yl)propan-2-amine hydrochloride) is a phenothiazine derivative (Figure 1). It is a first-generation H1 receptor antagonist, antihistamine, and antiemetic medication and can also have strong sedative effects [5]. Promethazine affects ligand gated ion channels such as purinergic P2 or cholinergic ACh receptors and voltage dependent ion channels such as sodium, calcium, or potassium channels. Beside, these effects, promethazine also inhibits the brain Na*þ*K*þ*-ATPase and the mitochondrial permeability transition pore. Since its first introduction in 1946, it has been used for prevention and treatment of nausea and vomiting caused by narcotic therapy, migraine episodes, cancer chemotherapy, and so forth. [6]. Meanwhile, it has become apparent that this drug interacts with many different receptors. It works by changing the actions of chemicals in brain and as an antihistamine [6]. It is used to treat allergic symptoms such as itching, runny nose, sneezing, itchy or watery eyes, hives, and itchy skin rashes, also to prevent motion sickness, and treat to nausea and vomiting pain after surgery and as a sedative or sleep aid, especially for oncologic patients. The function of PM is to block histamine H1 receptors without blocking the secretion of histamine. In therapeutic doses, CNS depression manifested by sedation is a frequent occurrence. The peripheral H(1)-receptor (PrH1R) stimulation leads to allergic symptoms while the brain H(1)-receptor (BrH1R) blockade leads to somnolence, fatigue, increased appetite, decreased cognitive functions (impaired memory and learning), seizures, aggressive behaviour, and so forth. First-generation oral AHs (FGAHs) additionally have potent antimuscarinic, anti-*α*-adrenergic, and antiserotonin effects leading to symptoms such as visual disturbances (mydriasis, photophobia, and diplopia), dry mouth, tachycardia, constipation, urinary retention, agitation, and confusion. The somnolence caused by FGAHs interferes with the natural circadian sleep-wake cycle, and therefore FGAHs are not suitable to be used as sleeping pills. Second-generation oral AHs (SGAHs) have proven better safety and tolerability profiles, much lower proportional impairment ratios, with at least similar, if not better, efficacy, than their predecessors. Only SGAHs, and specially those with a proven long-term (e.g., ≥12 months) clinical safety, should be prescribed for young children. Evidence exists that intranasally applied medications, like intranasal antihistamines, have the potential to reach the brain and cause somnolence. Second-generation oral antihistamines are the preferred first-line treatment option for allergic rhinitis and urticarial, although topical first-generation antihistamines are still prescribed by general healthcare practitioner and pharmacists too easily, without taking into account possible side effects as a first line treatment for several dermatological problems [7–12]. 

## 3. Absorption, Distribution, Metabolism, Elimination

PM is well absorbed from the gastrointestinal tract. Peak plasma concentrations occur after 2 to 3 hours when promethazine is administered orally (25 to 50 mg) or intramuscularly (25 mg). Following rectal administration of promethazine in a suppository formulation, peak plasma concentrations were observed after about 8 hours. Oral bioavailability is approximately 25%. Rectal bioavailability has been reported at 23%. Promethazine is widely distributed in body tissues and has a large apparent volume of distribution following oral and intramuscular administration. Promethazine has been reported to be 93% protein-bound when determined by gas chromatography and as 76 to 80% protein-bound when determined by HPLC. Promethazine rapidly crosses the placenta, appearing in the cord blood within 1.5 minutes when given intravenously at term. Promethazine crosses the blood brain barrier. The elimination half-life of promethazine following oral administration has been estimated to be within the range of 12 to 15 hours. After intravenous administration of 12.5 mg, blood concentrations of promethazine declined bioexponentially with a terminal elimination half-life of 12 hours [9]. PM is metabolized principally to promethazine sulphoxide and to a lesser degree desmethylpromethazine. The major site of metabolism is the liver and that the drug is subjected to extensive first-pass hepatic biotransformation, explaining the oral bioavailability of 25%. Metabolism also occurs in the gut wall but to a lesser degree than earlier postulated. The sulphoxide metabolite has not been detected after intramuscular dosing as circulating levels are probably below analytical detection limits due to a combination of slow absorption, lower dose (50% of oral), and bypass of first-pass metabolism in the liver [7]. Its elimination is primarily due to hepatic metabolism. No evidence was found to suggest that metabolites of promethazine are pharmacologically or toxicologically active. Promethazine has not been reliably detected in breast milk [8]. 

## 4. Clinical Use

PM is used for the treatment of allergic symptoms, often given at night because of its marked sedative effects. Drug hypersensitivity reactions and allergic conditions have also been treated with promethazine especially in emergencies. It can also be used in treating symptoms of asthma, pneumonia, or other lower respiratory tract infections; in fact, inhalation therapy for relieving bronchial spasm is made by quaternary salts of promethazine. PM is sometimes used for its sedative effects and in some countries is marketed for this purpose, including the sedation of young children as nasal sleep introducing drug, or it can be used as an anaesthetic premedication to produce sedation, reduce anxiety, or to reduce postoperative nausea and vomiting as dose-controlled transdermal device. The drug is often given in conjunction with an opiate analgesic such as pethidine, particularly in obstetrics. Taken before travelling, promethazine is effective in preventing motion sickness. Vomiting from other causes can be treated with higher or more frequent doses. It can be found in cough-relieving medicament as nonsteroidal glucocorticoid inhibitor for treating inflammation, allergy, and autoimmune conditions, for the reduction of intracranial pressure in particular for the prevention and treatment of elevated intracranial pressure and/or secondary ischaemia, caused in particular by brain injury. Dose-controlled transdermal promethazine compositions are used to provide antiemetic and antipruritic relief to patients, with the aim of minimizing side effects and adverse reactions known to occur with other routes of administration and other formulations. Combination of histamine H1R and H4R antagonist is used for the treatment of neoplastic disorders, consisting in a cytotoxic agent as an agent to prevent multidrug resistance [13]. It is also used as a contraceptive killing sperm in vagina, since promethazine hydrochloride has strong sperm-killing effect, or as an antimutagenic treatment of bacteria by killing bacteria. Bathing preparation, which contains a histamine H1-antagonist, inhibits the decomposition of hyaluronic acid, playing an important role in moisture and tension of skin to improve roughened or dried skin. This cosmetic can take such a form as gel, cream, spray, cataplasm, lotion, pack, milky lotion, or powder. It can also be a melanogenesis-suppressing agent useful as a skin-beautifying cosmetic, a skin-aging prevention agent, and so forth, by using a phenothiazine compound having remarkable melanogenesis-suppressing effect. Application of PM can be used for treating haemorrhoids with no pain, no side effect, no operation, and no hospitalization, but low cost [14–16]. Promethazine has been used to control extrapyramidal disorders in children caused by metoclopramide and levodopa-induced dyskinesia in patients with Parkinson's disease. In young children undergoing dental procedures, it has been suggested that promethazine can be used in conjunction with chloral hydrate to produce sedation, as there was observed a lower incidence of nausea than when chloral hydrate was administered alone. In some countries, promethazine is available as a 2% cream without medical prescription for the treatment of allergic skin conditions, insect bites, and burns; however, topical use is not recommended due to skin sensitization reactions. All these pharmacological properties contribute to the various therapeutic indications and side effects (Table 1).

## 5. Pharmacology and Toxicology

### 5.1. Toxicodynamics

The pharmacology of promethazine is complex, and for this reason toxicological mechanisms are not completely understood. Most reference texts suggest that the toxicity of promethazine is mainly due to its anticholinergic actions at muscarinic receptors. Many of the signs and symptoms of poisoning are similar to those observed with atropine. In the presence of anticholinergic effects, serious manifestations such as seizures, hallucinations, hypertension, and arrhythmias have been reversed by the administration of physostigmine. Besides anticholinergic 5 effects, promethazine can also exhibit toxic effects typical of antipsychotic phenothiazines. Hypotension and extrapyramidal signs may be attributable to antidopaminergic actions of promethazine [17]. 

### 5.2. Pharmacodynamics

Promethazine is a phenothiazine antihistamine, antagonizing the central and peripheral effects of histamine mediated by histamine H1 receptors. The drug does not antagonize histamine at H2 receptors. Antihistamines competitively antagonize most of the smooth muscle stimulating actions of histamine on the H1 receptors of the gastrointestinal tract, uterus, large blood vessels, and bronchial muscle. Increased capillary permeability and oedema formation, flare, and pruritus, resulting from actions of histamine on H1 receptors, are also effectively antagonized. Promethazine appears to act by blocking H1 receptor sites, preventing the action of histamine on the cell. Promethazine rapidly crosses the blood brain barrier and it is thought that the sedative effects are due to blockade of H1 receptors in the brain. Promethazine is not used clinically for its antipsychotic properties but in common with other phenothiazines exhibits antidopaminergic properties. The antiemetic effect of promethazine may be due to blockade of dopaminergic receptors in the chemoreceptor trigger zone (CTZ) of the medulla. Promethazine has strong anticholinergic properties, blocking the responses to acetylcholine that are mediated by muscarinic receptors. These atropine-like actions are responsible for most of the side effects observed in clinical use of the drug. Promethazine also has antimotion sickness properties which may be due to central antimuscarinic action. In concentrations several times higher than those required to antagonize histamine, promethazine exhibits local anaesthetic effects. Promethazine has also been shown to inhibit calmodulin. Authors have suggested that calmodulin inhibition by promethazine could be a mechanism involved in the blockade of histamine secretion at cellular level [18]. 

### 5.3. Toxicity

In adult human subjects minimum lethal exposure, and maximum tolerated exposure have not been clearly defined, mainly due to the lack of data on the exact amount ingested in cases of overdosage. Peak plasma levels following therapeutic oral doses of 30 to 50 mg in adults have ranged from 11 to 23 ng/mL. Adverse effects following intramuscular injection were associated with plasma levels of 48 ng/mL. In children promethazine is readily available in syrup form which is often administered to sedate young children. It is likely that in many cases the dose is excessive, leading to symptoms of toxicity. CNS toxicity with survival has been reported in children aged 5 to 12 years after accidental ingestion of 200 to 500 mg of promethazine (12.5 to 28 mg/kg). Death was reported in a two-year-old child with a history of ingesting 200 mg of promethazine as tablets [19]. 

### 5.4. Main Adverse Effects

Side effects usually reported are severe breathing problems or death in child younger than 2 years old. In adults, overdosage is usually characterized by CNS depression resulting in sedation and coma sometimes followed by excitement. In young children, CNS stimulation is dominant; symptoms include excitation, hallucinations, dystonias, and occasionally seizures. Anticholinergic manifestations such as dry mouth, mydriasis, and blurred vision are usually present. Overdosage may also present with various cardiorespiratory symptoms such as respiratory depression, tachycardia, hypertension or hypotension, and extrasystoles. Sedation, ranging from mild drowsiness to deep sleep, is probably the most common adverse effect. Dizziness, lassitude, disturbed coordination, and muscular weakness have all been reported. Gastrointestinal effects including epigastric distress, nausea, diarrhea, or constipation can occur. Promethazine can also cause immunoallergic reactions. Leucopenia and agranulocytosis have occurred rarely and usually in patients receiving promethazine in combination with other drugs known to cause these effects. Jaundice and thrombocytopenic purpura have been reported rarely. Extrapyramidal effects can occur, especially at high doses. Venous thrombosis has been reported at the site of intravenous injections. Arteriospasm and gangrene may follow inadvertent intra-arterial injection. Respiratory depression, sleep apnoea, and sudden infant death syndrome (SIDS) have occurred in a number of infants or young children who were receiving usual doses of promethazine [19–23]. 

### 5.5. Skin Exposure

Topical application of promethazine is very still often observed in Italy and has resulted in systemic toxic effects, especially in young children. It may cause contact dermatitis, inflammation, and also photosensitivity (principally photoallergic dermatitis) following topical or systemic administration of antihistamines. Acute or chronic urticaria has been reported following oral ingestion. The reaction may appear as eczema, pruritic, papular rash, or erythema. In cases of fixed drug eruption (FDE), skin lesions recur at the same sites after repeated exposure in 1-2 weeks, and the interval between reexposure and eruption appearance is usually 24 h. All body sites can be involved. Although cross-reactivity to chemically related drugs has often been reported, only a few cases of photoallergic reactions due to two or more unrelated agents have been described. Promethazine hydrochloride is an H1 antihistamine derived from phenothiazine. Although promethazine methylenedisalicylate is very widely and frequently used, documented reports of drug eruptions, in particular FDE, in response to the H1 antihistamine included in this medicine, are rare. The explanation may be that this drug has a relatively large adjacent structure that includes a benzene ring [24, 25].

### 5.6. Diagnosis

The diagnosis of drug allergy requires a thorough history and the identification of physical findings and symptoms that are compatible with drug-induced allergic reactions. Depending on the history and physical examination results, diagnostic tests such as skin testing, graded challenges, and induction of drug tolerance procedures may also be required. Therefore, if drug allergy is suspected, evaluation by an allergist, experienced in these diagnostic procedures, is recommended. It originates on the applied site and eventually spread. Urticaria (hives) and angioedema (swelling) are also common and can result from both IgE-mediated and non-IgE-mediated mechanisms. The most severe forms of cutaneous drug reactions are Stevens-Johnson syndrome (SJS) and toxic epidermal necrolysis (TEN). Given the severity of these conditions, drugs suspected of causing SJS and TEN should be strictly avoided by the patient in the future. Although skin reactions are the most common physical manifestation of drug-induced allergic reactions, many other organ systems may be involved such as the renal, hepatic, and hemolytic systems. Antihistamine poisoning must always be considered in patients presenting with a central anticholinergic syndrome. Concentrations of promethazine in blood and urine can be determined [26–29].

### 5.7. Diagnostic Tests

Skin testing procedures, such as skin prick testing (SPT) and intradermal tests (test in which the allergen is injected into the skin dermis), are useful for the diagnosis of IgE-mediated (type I) reactions. Positive skin tests to these drugs confirm the presence of antigen-specific IgE and support the diagnosis of a type I hypersensitivity reaction. With some agents, a negative skin test does not effectively rule out the presence of specific IgE. Serum-specific IgE tests are available for a limited number of drugs. However, these tests are costly and generally less sensitive than skin tests. Furthermore, most of these in vitro tests are not adequately validated for drug allergy testing. Patch testing involves placing potential allergens (at nonirritant concentrations) on the patient's back for 48 hours under aluminum discs and then assessing for reactions. Drug patch testing is useful for the diagnosis of various delayed (type IV) cutaneous reactions. The measurement of histamine and tryptase levels has proved useful in confirming acute IgE-mediated reactions, particularly anaphylaxis; however, negative results do not rule out acute allergic reactions. A complete blood count can help diagnose hemolytic (type II) drug-induced reactions, such as hemolytic anemia, thrombocytopenia, or neutropenia. Hemolytic anemia may also be confirmed with a positive direct and/or indirect Coombs' test (used to examine the presence of antibodies on red blood cell membranes). In cases where there is a definite medical need for a particular drug, but the clinical diagnosis of drug allergy remains uncertain despite thorough investigations, a procedure to induce temporary drug tolerance (also referred to as drug desensitization) or graded challenge testing (also known as provocation testing) may be considered. Induction of drug tolerance procedures temporarily modifies a patient's immunologic or nonimmunologic response to a drug through the administration of incremental doses of the drug. These tests are generally used to determine whether a patient will have an adverse reaction to a particular drug, by administering subtherapeutic doses over a period of time while observing the patient for potential reactions. They are not advised if the patient has experienced a previous life-threatening reaction to the drug in question. Drug tolerance-induction procedures and graded challenges are potentially harmful and should only be performed by experienced personnel in facilities with resuscitative equipment readily available [29–33].

## 6. Clinical Features and Diagnostic Aspects of PM Allergic Reactions

**Figure 2:**
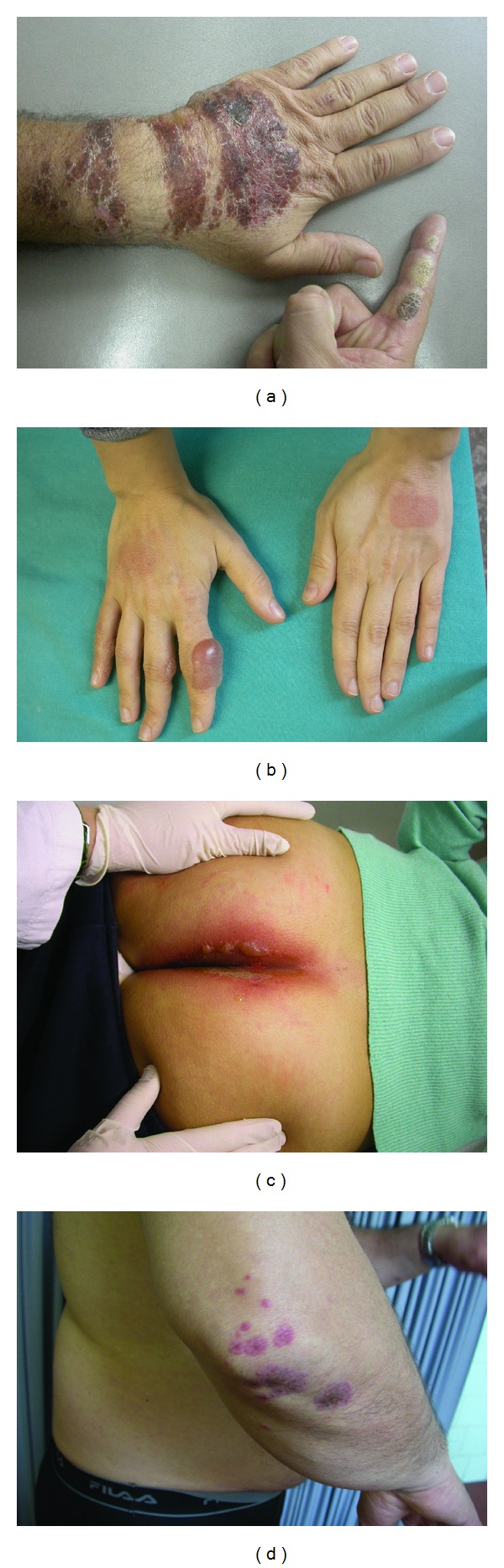
(a) Acute dermatitis with edema, erythema, bullous lesion at the application site of topical PM of the left hand. (b) Erythematobullous eruption of the hands after topical application of gel after sun exposure. (c) Mucous bullous involvement after systemic absorption of PM. (d) Urticaria after systemic absorption of PM.

**Figure 3:**
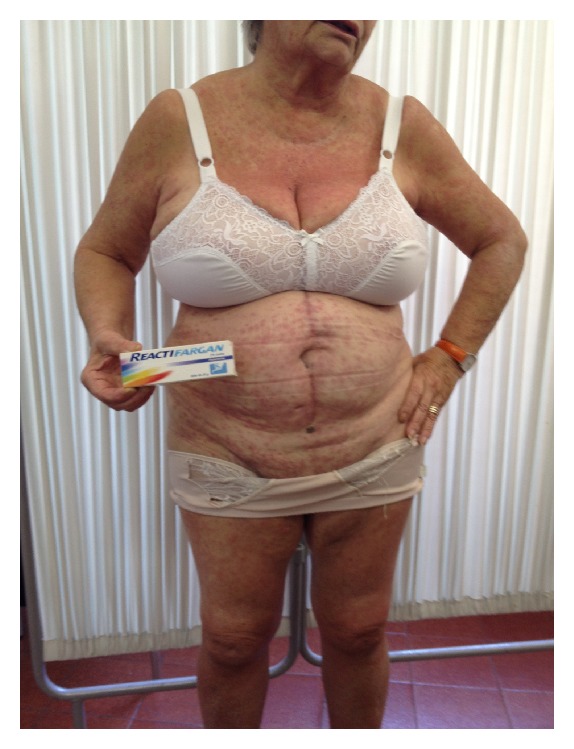
Systemic maculo-papular eruption associated with urticarial rash after topical application of PM for insect bite and solar exposure.

In our experience photocontact dermatitis due to topical promethazine is the most frequent reactions observed in almost 15% of patients and develops as an acute dermatitis with edema, erythema, papulovesicles, bullous, itching lesions, or erythema exsudativum multiforme-like eruption (Figures 2(a), 2(b), 2(c), and 2(d)) at the application site 1 week to 1 month after the initiation of use, depending on the frequency and intensity of sun exposure. The lesions may be apparently confined to the body sites, such as elbows, knees, ankles, forearms, and thighs, or several parts of the body may be affected where there are insect bites or burns, and sun exposure concomitantly occurs, but may also appear on other sites by transfer (ectopic contact dermatitis by hands or clothing; it may also affect family members due to connubial contact). Moreover, PM contaminates clothing, shoes, and so forth, explaining some of the persistent reactions. Most of them presented with several types of skin lesions. Sometimes patients need systemic treatment with corticosteroids. Emergency visits in the hospital may occur. Cessation of the causative agents and avoidance of sun exposure in combination with topical application of glucocorticosteroids or zinc oxide usually improve the symptoms in 2 weeks. However, residual postinflammatory hyperpigmentation may occur and in a rare instance. In subjects previously sensitized to PM, the systemic absorption of these drugs through multiple routes of administration (oral, parenteral, or topical) can induce the so-called systemic contact dermatitis, which can present as generalized maculapapular, papulovesicular, pustular, or erythematous eruption as well as urticarial rash (Figure 3). Clinical suspicion should be confirmed by patch testing such as SIDAPA (Società Italiana di Dermatologia Allergologica Professionale ed Ambientale) patch test standard series, including fragrance mix and its components (eugenol, isoeugenol, oak moss, geraniol, hydroxycitronellal, amylcinnamaldehyde, cinnamyl alcohol, and cinnamaldehyde) and with the SIDAPA photopatch test series. Patch tests, in patients with contact sensitization to these agents, can evoke false negative reactions because of the intrinsic antihistamine action of Promethazine which may suppress or delay the cutaneous response. Therefore, reading should be postponed on day 5 or 7. At the first reading, the reaction may appear only at the edges of the test area, while it can be completely absent in its central portion, where the antihistamine effect of promethazine is more evident because of the accumulation at higher concentrations. This phenomenon named “edge” or “border effect” fades away on successive readings after a few days.

## 7. Mechanisms Underlying Sensitization to Promethazine

Photoallergy is due to a cell-mediated hypersensitivity response, involving immunological reactions. Therefore, it only occurs in previously sensitized individuals and requires a latency period of sensitization and allergic contact dermatitis to prometazine. Photosensitization, phototoxicity, or photoallergy is induced on exposure to sunlight after internal or external administration of promethazine. Antihistamines are absorbed via the gastrointestinal tract and are carried to the near surface of skin by the circulation, where it is directly exposed to sunlight. In general, photoreactions by photosensitive chemicals are divided into the phototoxic and photoallergic types. While phototoxicity is mediated by active oxygen, especially singlet oxygen, photoallergy occurs as a consequence of a specific immune reaction mediated by antigen-specific sensitized T cells. It is clinically well known that topical promethazine induces photocontact dermatitis as an adverse reaction. When PM is used by patients (through ingestion or skin contact), Langerhans cells in the epidermis process them and display them in a complex with HLA-DR. This is presented to an LTCD4, interaction with the T-cell receptor-CD3 complex occurs, and the T allergen is recognized. This leads to a proliferation and recruitment of lymphocytes with release of vasoactive substances and direct inflammatory mediators. In this process, DNA damage is present and often caused by an oxidative stress. The sensitization to PM is often a cross-reaction to other drugs of the family of phenothiazine. In the reactions to Phenothiazine drugs, a phototoxic component may be present. Prometazine has both phototoxic and photoallergic potentials, but many clinical observations have indicated that photosensitivity to MP is a photoallergic reaction. In fact, promethazine in vivo test, demonstrated relatively weak phototoxicity. The major photoproduct elicits photoperoxidation and causes red blood cell photohemolysis. Promethazine may induce DNA damage in vitro upon irradiation. DNA, in the presence of promethazine, undergoes single strand breaks involving hydroxyl radicals. Free radicals were reported to damage DNA and to induce hemolysis and active oxygen species. Superoxide anion and singlet oxygen generated from ultraviolet (UV)-exposed promethazine were found to contribute to dermatitis. The two theories, named photohapten and prohapten models, have been put forward to explain the formation of photo-allergen. According to the photohapten theory, photosensitizing chemicals and protein need to coexist upon exposure to UVA in a noncovalent manner and UVA turns it covalent. On the other hand, the prohapten theory suggests that UVA simply converts photosensitizing substances into ordinary hapten, which subsequently binds to protein. It seems that most of the photoallergic substances have a photohaptenic moiety; promethazine serves as a photohapten because of its photocoupling ability to protein. This contradiction is due to the limitation of the speculation from the clinical data. Patients had different personal histories using several kinds of drugs. They may have used the photoreacted drugs independently which means there was the possibility of an independent photosensitization [34, 35]. 

## 8. Cross- and Concomitant Reactivity

Many topical photoallergic culprits have been reported in the literature, the most important of which are sunscreen agents and, recently, diuretic, and antibacterial nonsteroidalantiinflammatory agents (NSAIDs). Not at all exceptional is the occurrence of photoaggravation and recurrent transient or even persistent light reactions on previously exposed as well as nonexposed areas (often sparing the original application site), particularly with the topical antihistamines. Moreover, cross-reactions with chemically related as well as nonchemically related molecules are common. Photo sensitizing chemicals absorb ultraviolet (UV) and/or visible radiation, a characteristic that is essential for the chemical to be regarded as a photosensitizer. In order to elucidate the antigenic determinant in PM-induced photoallergy, studies on cross-reaction with structurally related compounds are important. PM can be found in association with NSAIDs or other drugs. Cross-sensitivity reactions with other arylpropionic acid derivatives, such as tiaprofenic acid, fenofibrate, or oxybenzone-harbouring benzoyl ketone or benzophenone, may also occur. According to the other ingredients in the PM-containing transdermal delivery (which may contain folium eriobotryae, pericarpium papaveris, radix stemonae, cynanchum glaucescens, the root bark of white mulberry, *platycodon grandiflorum*, menthol crystal, cane sugar, citric acid, essence, sodium benzoate, dextromethorphan hydrobromide, promethazine hydrochloride, or 75% of alcoholic solution), some patients may react with a contact allergic reaction to other components in the gel, such as lavender oil which is a natural fragrance. Linalool, which is a major ingredient in lavender oil, is a sensitizer when oxidized. Cinnamyl alcohol, that is, a component of both fragrance mix and *Myroxylon pereirae* has been suggested to explain this phenomenon. In patients with contact allergy to PM, concomitant positive reactions to cinnamyl alcohol are due to cross-sensitization, whereas simultaneous allergic reactions to fenticlor, octocrylene, and benzophenone-10 should be regarded as cosensitizations [36, 37].

## 9. Risk Factors

Factors associated with an increased risk of developing a drug allergy include age, gender, genetic polymorphisms, certain viral infections, and drug-related factors (e.g., frequency of exposure, route of administration, and molecular weight). Drug allergy typically occurs in young and middle-aged adults and is more common in women than men. Genetic polymorphisms in the human leukocyte antigen (HLA; a gene product of the major histocompatibility complex) as well as viral infections, such as human immunodeficiency virus (HIV) and the Epstein-Barr virus (EBV), have also been linked to an increased risk of developing immunologic reactions to drugs. Susceptibility to drug allergy is influenced by genetic polymorphisms in drug metabolism. In addition, topical, intramuscular, and intravenous routes of administration are more likely to cause allergic drug reactions than oral administration, while intravenous administration is associated with more severe reactions. Prolonged high doses or frequent doses are more likely to lead to hypersensitivity reactions than a large single dose. Although atopic patients do not have an increased risk for drug allergy, they are at increased risk for serious allergic reactions [38].

## 10. Management

Symptomatic supportive therapy is indicated and general measures such as maintenance of adequate ventilation and cardiovascular function must be instituted if necessary. Gastric emptying may be successful even if delayed for up to 2 hours. Emesis should probably not be induced due to the risk of coma or psychosis developing in the patient. Administration of activated charcoal would be preferred. The use of a cathartic is no longer recommended. In the absence of seizures, gastric lavage (with endotracheal tube with cuff inflated in place to prevent aspiration of gastric contents) may be beneficial. Seizures may be controlled with intravenous diazepam (preferred) or phenytoin. Unless severe, hypotension should be treated with posture; severe cases can be treated with fluids or pressor agents. In severe hypertension, parenteral sodium nitroprusside may be required. Dystonic reactions frequently respond to intravenous diphenhydramine. In the presence of severe anticholinergic effects, physostigmine, by slow intravenous injection, has been administered. However, the use of physostigmine is considered controversial [39]. 

## 11. Discussion 

The topical use of PM, widely used for moderate acute and chronic itching conditions, is one of several strategies used to improve the tolerability profile of PM, particularly with regard to gastric and renal adverse effects. However, topical can induce photosensitivity. Among the different topically uses, PM has often been implicated in photosensitivity reactions. The higher frequency of such adverse reactions could be accounted for by its chemical structure and the variety of chemical reactions that give rise to the phototoxic effects. PM allergy encompasses a spectrum of immunologically mediated hypersensitivity reactions with varying mechanisms and clinical presentations. This type of adverse drug reaction (ADR) not only affects patient's quality of life, but may also lead to delayed treatment, unnecessary investigations, and even mortality. Given the myriad of symptoms associated with the condition, diagnosis is often challenging. Therefore, referral to a dermatologist or an allergist experienced in the identification, diagnosis, and management of drug allergy is recommended if a drug-induced allergic reaction is suspected. Diagnosis relies on a careful history and physical examination. In some instances, skin testing, graded challenges, and induction of drug tolerance procedures may be required. The most effective strategy for the management of drug allergy is avoidance or discontinuation of the offending drug and avoidance of sun exposure. When available, alternative medications with unrelated chemical structures should be substituted. Cross-reactivity among drugs should be taken into consideration when choosing alternative agents. Additional therapy for drug hypersensitivity reactions is largely supportive and may include topical corticosteroids, topical zinc oxide in severe cases, and systemic corticosteroids. In the event of anaphylaxis, the treatment of choice is injectable epinephrine. Promethazine ARs are classified as either predictable reactions that may occur in anyone or unpredictable reactions that occur in only susceptible individuals. Predictable reactions are the most common type of ADR and are usually dose dependent and related to the known pharmacologic actions of the drug (e.g., side effects, overdose, and drug interactions). Unpredictable reactions occur in approximately 20–25% of patients who experience ADRs; these reactions are generally unrelated to the pharmacologic actions of the drug. Promethazine ARs are common in our daily dermatological clinical practice, affecting between 15 and 25% of patients; serious reactions occur in 7% of patients. In summary, is a potent photosensitizer with many simultaneous photocontact allergies to various photosensitizers, some of which have structural similarities. The widespread and repeated use of these agents may lead to sensitization, incurring a greater risk of systemic allergic reactions with oral or other drugs, recognized to induce cross-reactions. Physicians and pharmacists should advise patients and inform them of the risks of their topical use which are often dispensed as over-the-counter drugs. Adverse photosensitivity responses to drugs occur predominantly as a phototoxic reaction which is more immediate than photoallergy and can be reversed by withdrawal or substitution of the drug. The bias and inaccuracy of the reporting procedure for these adverse reactions is a consequence of the difficulty in distinguishing between sunburn and a mild drug photosensitivity reaction, together with the patient being able to control the incidence by taking protective action. Prevention of photosensitivity involves adequate protection from the sun with clothing and sunscreens. In concert with the preponderance of free radical mechanisms involving the photosensitizing drugs, some recent studies suggest that diet supplementation with antioxidants may be beneficial in increasing the minimum erythemal UV radiation dose. Patients with photoallergy should avoid using some, such as suprofen or tiaprofenic acid, and the antilipidemic. The history is often not good guidance to determine related (photo)allergic contact dermatitis and the severe clinical symptoms sometimes require hospitalization and/or systemic corticosteroids. As for the association between and sunscreen intolerance (being 1 of the possible causal factors for recurrent dermatitis), routine standard photopatch testing might be indicated. We concluded that topical medicaments containing PM should not be used on exposed areas during spring and summer.

## 12. Prevention of Future Reactions

Prevention of future reactions is an essential part of patient management. The patient should be provided with written information about which drugs to avoid (including over-the-counter medications). The drugs should be highlighted in the hospital notes and within electronic records (where available) and the patient's family physician should be informed of the drug allergy. Engraved allergy bracelets/necklaces, such as those provided by MedicAlert, should also be considered, particularly if the patient has a history of severe drug-induced allergic reactions.

## 13. Conclusions

Because of the widespread nature of the problem, it is important to keep primary healthcare and pharmacist aware of the problem. Irrational drug therapy with prescribing errors was apparent in primary care practice, which may be related to a lack of drug information, pharmacovigilance programme, and nonadherence to basic principle of prescribing. In 2009 FDA reiterated the problem of promethazine tissue damage including suggestions to prevent harm and announced that it is now requiring manufactures to include a boxed warning about risk of serious tissue damage for iv injection; it would be useful to increase awareness also about the skin reactions which are too often underestimated. If possible, remove PM from the formulary or alert pharmacist to avoid to give it without a medical prescription, use alternatives when appropriate, and in case of reactions call a dermatologist promoting a rational drug use. The purpose of this paper has been to educate healthcare professionals who may have not been aware of the problem. Most importantly we outlined numerous steps that should be taken to minimize the potential for adverse outcomes. Since promethazine allergy is becoming a common clinical problem, assessment by a dermatologist or an allergist is important for appropriate diagnosis and management of the condition but especially for improving prescribing habits and incorrect information.

## Figures and Tables

**Table 1:** PM clinical use.

Common use	Other less common uses
Drug hypersensitivity reactions Allergic conditions in emergencies Asthma, pneumonia Other lower respiratory tract infections Nasal sleep-introducing drug Anaesthetic premedication, particularly in obstetrics To reduce postoperative nausea and vomiting particularly in obstetrics To prevent motion sickness As a cough-relieving medicament for treating inflammation, allergy, and autoimmune conditions For the reduction of intracranial pressure Antipruritic relief to patients For the treatment of neoplastic disorders Sperm-killing effect To inhibit the decomposition of hyaluronic acid As a melanogenesis-suppressing agent For treating haemorrhoids	To control extrapyramidal disorders in children To produce sedation in dental procedures in children As a 2% cream for allergic skin conditions, insect bites, and burns
